# Life course socioeconomic status, chronic pain, and the mediating role of allostatic load: findings from the midlife in the United States

**DOI:** 10.3389/fpubh.2024.1365105

**Published:** 2024-03-18

**Authors:** Yunlong Liang

**Affiliations:** Institute for Social and Economic Research, University of Essex, Colchester, United Kingdom

**Keywords:** life course, chronic pain, allostatic load, chain of risk additive model, midlife adults

## Abstract

**Introduction:**

Low socioeconomic status (SES) has been linked to chronic pain (CP); however, the mechanisms by which SES over the life course influences downstream CP outcomes remain unclear.

**Methods:**

This study utilizes data from the Midlife in the United States (MIDUS) survey, a prospective sample of community-dwelling individuals (*N*=781), to investigate the chain of risk additive model of SES in relation to CP. Additionally, the study examines the mediating role of allostatic load (AL) in the relationship between life course SES and CP. Confirmatory factor analysis was employed to capture the multidimensionality of life course SES and path analysis was used to examine the direct and indirect effects on CP. AL was computed by quartile-based summation and by latent class analysis.

**Results:**

Results indicated lower SES in MIDUS 2 was associated with greater high-interference CP odds in MIDUS 3 (OR=1.069, 95% CI=1.006-1.136, *P* < 0.05) and no association was found between distal SES and levels of CP interference. Similarly, no significant relationship was observed between SES and the number of CP locations. Additionally, no additive effects of SES were found, and AL did not present mediation effects on the association between life course SES and CP.

**Discussion:**

The present study emphasizes the importance of directly proximal effects of SES on CP, underscoring the need for equitable distribution of health resources and the implementation of policies focused on diminishing socioeconomic inequalities. Further research is needed to examine alternative pathways by which proximal SES impact CP.

## 1 Introduction

Chronic pain (CP) is pain that lasts or recurs for more than three months (1). In 2019 in the United States, over fifty million adults experienced CP and twenty-four million people endured work and life limitations brought about by high-impact pain (2). This prevalence of CP has been on an increasing trend (3, 4). CP not only impairs an individual’s health and economic status but also affects social relationships (5). Additionally, it imposes a significant burden on the public health system and diminishes societal productivity (2). Individuals’ socioeconomic status (SES) is recognized as a fundamental cause of chronic diseases (6, 7). CP exhibits a disproportionately distribution across different SES groups (8). However, there is a paucity of research exploring the association between life course SES and the odds of having CP, as well as the underlying mechanisms mediating this relationship.

### 1.1 Background

#### 1.1.1 Life course SES and CP

Life course epidemiology proposes several interrelated models for the origin of chronic diseases. Firstly, there may exist a critical or sensitive period during which risk exposures occurring in this window may have significant impacts on downstream health outcomes (9). Notably, critical periods emphasize the plasticity of biological development, especially in childhood, where risk exposure can irreversibly alter physiological structures (10), and these changes independently affect health outcomes. Risk exposures during a sensitive period may have a greater impact on health outcomes than at other times (9), but do not involve permanent biological changes.

Besides, life course SES involves influence pathways, where early SES directly or indirectly affects subsequent SES, thereby jointly influencing the risk of later diseases. Therefore, some scholars posit that the chain of risk additive model might better fit the transmitting effects of life course SES (11). The chain of risk additive model not only allows for the detection of the direct effects of exposures at different times but also for examining the mediating effects of later exposures and the cumulative effects in the risk chain (11).

Recent life course studies identified significant associations between SES indicators and CP (12, 13). Despite significant associations from other life course studies (14–16), the measures overlooked the duration of pain and its impact on daily life. Moreover, there was insufficient control for early confounders, especially for those relate to health selection of SES (17) and was a lack of consideration for downstream effects of previous SES on subsequent SES. Furthermore, the current study evaluated additive effects using a cumulative score, which assumed equivalent impacts of SES at different periods on CP.

Finally, single indicators were used as proxies for SES, which did not consider the covariance to comprise SES as a whole and might fail to capture the complex interplay between these SES indicators. Also, subjective SES is an often-overlooked yet unique dimension in measuring SES in CP research (8), as subjective SES reflects the socio-psychological coping resources people have in response to objective SES. Low subjective SES, such as perceived economic hardship and daily financial worries, is not only a stressor in itself but also limits capacity to manage stress (18, 19). Over time, individuals frequently experiencing low subjective SES may become more vulnerable to stressors, thereby increasing the likelihood of experiencing pain (19).

#### 1.1.2 AL as a potential mediator in the association between life course SES and CP

The current body of research on SES and CP lacks an examination of intermediary mechanisms and researchers have suggested dysregulated stress response could connect life course SES with CP (8). Allostatic load (AL) describes the biological consequences of an organism’s continuous adaptation to prolonged and repeated stress (20, 21). The biological cost of chronic stress initially manifests in alterations of the hypothalamic–pituitary–adrenal (HPA) axis. A normal HPA axis, through its reactive hormonal secretion, prepares the organism for stress response (22). However, prolonged activation of the HPA axis, leading to over-secretion of glucocorticoids and catecholamines, may eventually disrupt the production of substances necessary for maintaining the normal functioning of downstream physiological systems. This disruption can result in anomalies in biomarkers from multiple physiological systems (20).

Over the past decades, an abundance of research on SES and AL has emerged, consistently indicating that lower SES is associated with higher levels of AL (23, 24). Furthermore, CP correlates with chronic stress and may be accompanied by abnormalities in several biological systems. For instance, there is often dysfunction in the HPA axis, the autonomic nervous system, and the immune system among CP patients (25, 26). Given a high overlap in biological dysregulation between CP and AL, such as an inability to habituate to repeated stressors, failure to shut off stress responses, and altered or inefficient responses to stress, ultimately leading to increased compensatory responses at the cellular level to other mediators, some scholars suggest that CP may be a AL disease (27). Recent population-based studies have found a cross-sectional relationship between CP and AL (28–30). However, the predominant operationalization of AL is the summation method. This approach failed to differentiate driven pattern of AL and overlooked interaction between AL biomarkers. Latent Class Analysis (LCA) may offer a solution to these methodological shortcomings (31).

**Figure 1:**
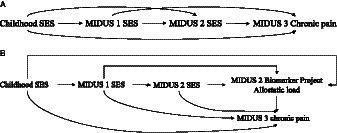
**(A)** Chain of risk additive model of SES. **(B)** Chain of risk additive model of SES with AL.

The primary aim of this study was to examine the prospective association between SES and CP by integrating SES across the life course into the chain of risk additive model. Therefore, the study employed path analysis to detect the direct effects of SES on prospective CP within the chain of risk additive model, the indirect and additive effects of SES at different periods (see Figure 1A), and the mediating effect of AL (see Figure 1B).

## 2 Methods

### 2.1 Data

**Figure 2:**
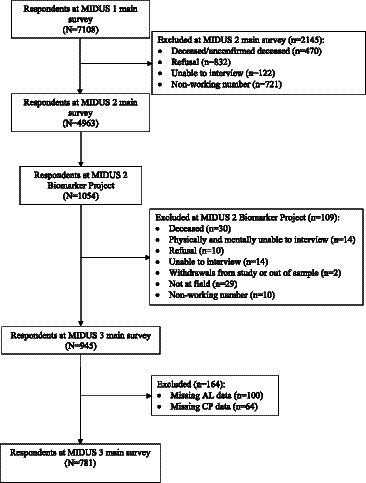
Flowchart for eligible sample.

This research utilized data from the Midlife in the United States (MIDUS) study. It encompassed three primary survey waves and a biomarker project. The MIDUS main survey is a national longitudinal study on individual social status, psychological profiles, and biological processes of aging, begun between 1995–1996 and followed 7,108 non-institutionalized Americans aged 25 to 74 in the contiguous United States. The MIDUS 2 and MIDUS 3 main surveys followed the original respondents and collected data through phone interviews and self-administered questionnaires between 2004–2006, and 2013–2014, respectively. A total of 1,255 respondents participated in the Biomarker Project of MIDUS 2 conducted from 2004 to 2009 with 201 non-probability samples of the Milwaukee project. Samples meeting the subsequent criteria were incorporated into the final analysis: (1) samples that completed one baseline survey, two MIDUS follow-up surveys and participated in the biomarker program; (2) samples that provided complete information on the AL and CP information (Details in Figure 2). The MIDUS is publicly accessible secondary data, and both verbal consent and written consent were obtained from the participants. More details of the study are available on the MIDUS website.^1^

### 2.2 Measures

#### 2.2.1 Dependent variable: chronic pain in MIDUS 3

Assessing the level of pain interference and the number of CP sites, as opposed to simply reporting the presence of CP, can provide valuable insights into the intensity of pain, its impact on daily activities, and the extent of pain distribution across the body, which are crucial for devising effective pain interventions (32, 33). Respondents were first asked “Do you have chronic pain, that is do you have pain that persists beyond the time of normal healing and has lasted from anywhere from a few months to many years?”; if so, they were then asked about CP interference. A pain interference index was generated by a mean score of how much pain interfered with respondents’ activity, mood, relations, sleep, and enjoyment, ranging from 0 to 10 (34). Then, the CP interference index was further categorized into no pain, low interference pain (≤4), and high interference pain (>4) as a categorical variable based on the Brief Pain Inventory Subscale cutpoint (35, 36). In addition, if respondents reported having CP, they were asked about the location of the pain, including head, neck, back, arms, legs, shoulders, hips, knees, and other sites. The pain sites were summed up to an index and then categorized it into no pain, 0–2 sites, or 3 or more sites as a categorical variable.

#### 2.2.2 Allostatic load – potential mediator

AL biomarkers were collected from the MIDUS 2 Biomarker Project. Followed by previous studies (20, 31, 37–39), AL was constructed by seven physiological systems and 27 biomarkers (shown in Table 1). High-risk quartiles of biomarkers were used to compute AL (40). DHEA-S and cortisol in the upper or lower 25^th^ quartiles were regarded as at high risk. When HFHRV, LFHRV, RMSSD, and SDRR strength fell within their lower 25^th^ quartile ranges, they were at high risk. Other biomarkers falling into their upper 25^th^ quartile were assigned to the high-risk range. Meanwhile, biomarkers in their high-risk quartiles were coded as 1; otherwise, 0. Then, an AL index was computed by summing up biomarker risk scores, which theoretically would range from 0 to 27.

**Table 1:** High-risk values for AL biomarkers.

Biomarker	High risk quartile
Hypothalamic pituitary adrenal axis	
DHEA-s (ug/dL)	≤51 or ≥ 141
Urine cortisol (μg/g)	≤6.7 or ≥ 19
Sympathetic nervous system	
Urine epinephrine (μg/g)	≥2.464
Urine norepinephrine (μg/g)	≥32.964
Urine Dopamine (μg/g)	≥182.964
Parasympathetic nervous system	
High-frequency HRV	≤55.9
Low-frequency HRV	≤103.4
RMSSD	≤12.02
SDRR (m s)	≤23.27
Cardiovascular	
Resting heart rate (bpm)	≥79.8
Resting SBP (mmHg)	≥144
Resting DBP (mmHg)	≥82
Metabolic-glucose	
Fasting glucose	≥105
Hemoglobin A1c%	≥6.242
HOMA-IR	≥4.36
Metabolic-lipids	
Triglycerides (mg/dL)	≥156
WHR	≥0.965
BMI	≥33.028
LDL cholesterol (mg/dL)	≥127
HDL cholesterol (mg/dL)	≤43
Inflammation	
CRP (mg/L)	≥3.655
IL6 (pg/mL)	≥1.23
TNF-α (pg/mL)	≥2.51
Fibrinogen (mg/dL)	≥399
E-Selectin (ng/mL)	≥51.88
ICAM-1 (ng/mL)	≥335.185
Blood Fasting IGF1 (Insulin-like Growth Factor 1) (ng/mL)	≥157

Then, LCA was used to capture the phenotypes of AL (package “poLCA” in R). The binary biomarkers were fitted into 1–7 clusters, and the selection of the optimum number of cluster was based on log-likelihood, Akaike Information Criterion (AIC), Bayesian Information Criterion (BIC), entropy, and interpretability of classification. Regarding entropy, an ideal value is close to 1, and above 0.8 is acceptable (41). As for AIC and BIC, lower values indicate a better fit (42). However, BIC tends to favor simpler models in larger samples due to its complexity penalty, while AIC may lean toward more complex models. Given these considerations, seeking points of inflection or plateauing for BIC and AIC can balance model complexity against the risk of overfitting (42). Also, the classification should be meaningful from a clinical or a biological perspective (42). 5,000 iterations were set to generate convergent estimation for each LCA model.

#### 2.2.3 Socioeconomic status – exposure of interest

The indicator selection and operationalization of SES were based on previous research (37, 43, 44). To refine the operationalization of SES, the study employed both subjective and objective SES indicators and measured SES through confirmatory factor analysis. There were three SES periods: childhood, adulthood in MIDUS 1 and MIDUS 2. Each SES indicator was recoded into an index ranging from 0 to 2, where 0 represented high SES conditions, 1 represented middle SES conditions, and 2 signified the low SES conditions (Details see Table 2).

**Table 2:** Operationalization for SES indicators.

SES indicators	SES categories and values assigned		
	0 (Least disadvantaged)	1 (Medium)	2 (Most disadvantaged)
Childhood SES (MIDUS 1, retrospective)			
Highest level of parental education (father’s and mother’s)	Bachelor’s degree or more	High school/GED/some college	Less than high school
Financial level growing up	1. A lot better off, 2. Somewhat better off, 3. A little better off	4. Same as average family	5. A little worse off, 6. Somewhat worse off, 7. A lot worse off
Father’s occupation (Census 1980 classification)^*^	1. Managerial and professional specialty occupations, 2. Technical, sales and administrative support occupations, 3. Service occupations	1. Operators, fabricators and laborers, 2. Farming, forestry and fishing occupations	1. Precision production, craft and repair occupations, 2. Experienced unemployed not classified by occupations
Adulthood SES (MIDUS 1 & MIDUS 2)			
Income-to-needs ratio adjusted for family size and year	Affluent/adequate-income	Low-income	Poor/extreme poverty
Highest level of education	Bachelor’s degree or more	High school/GED/some college	Less than high school
Rating of current financial situation	Best	Medium	Worst
Money to meet needs	More than enough money	Just enough money	Not enough money
Difficulty to pay monthly bills	Not at all difficult	Not very difficult/somewhat difficult	Very difficult
Occupation (Census 1980 classification for MIDUS 1, Census 1990 classification for MIDUS 2)^†^	1. Managerial and professional specialty occupations, 2. Technical, sales and administrative support occupations, 3. Service occupations	1. Operators, fabricators and laborers, 2. Farming, forestry and fishing occupations	1. Precision production, craft and repair occupations, 2. Experienced unemployed not classified by occupations

^*^Noting that if a father never worked due to disability, addiction, or mental issues, they were classified as unemployed. If they did not work for other reasons like raising children at home, the mother’s occupation represented the father’s when childhood occupation variable was constructed. ^†^Noting that if the current employment status of a respondent was unemployed, permanently disabled, never worked, or due to other reasons, they were classified as unemployed. If they were temporarily laid off, on maternity or sick leave, or retired, their occupation was represented by their last held position. If they were a homemaker or a part-time student, their spouse or partner’s occupation was represented. The occupation of a full-time student was represented by their father’s occupation.

#### 2.2.4 Covariates

The selection of covariates was based on previous literature and existing knowledge (12, 13, 28, 37).

##### 2.2.4.1 Time invariant variables

gender, ethnicity, whether the individual lived with an alcoholic and smoker during childhood, whether the individual lived with their biological parents, parental health.

##### 2.2.4.2 Time variant variables in MIDUS 1 and in MIDUS 2

age, marital status, physical activity index, smoking and drinking status, and multimorbidity. Physical activity index was also adjusted, computing by the moderate and vigorous activity index at MIDUS 1 and the light, moderate, and vigorous activity index at MIDUS 2. Smoking status was categorized into non-smokers, former smokers, and current smokers. Alcohol intake status was categorized into non-drinkers or those who rarely drink, light drinkers, and moderate or above drinkers. The chronic condition index (45) greater than 2 was coded as multimorbidity. Time-invariant variables were accounted for in each pair of associations, and both current and preceding wave confounders were adjusted for in the prospective analysis linking the current wave to a subsequent wave.

### 2.3 Statistical analyses

All analyses were conducted using R Studio ‘Lavaan’ package and structural equation modeling (SEM) was used. First, confirmatory factor analysis was employed to measure latent variables for SES and to assess the efficacy of the single SES indicators used in measuring SES as a whole. Then, path analysis was used to examine the chain of risk additive model. Full information maximum likelihood (FIML) method was applied to all models to handle missingness because it was found efficient under the assumptions of data being missing completely at random or missing at random (46). Additionally, the standard errors and chi-square test statistics in this method are robust to non-normality. A comprehensive assessment of each model fit can be achieved by simultaneously evaluating a set of specified indices. Generally, the comparative fit index (CFI) greater than 0.95 is considered acceptable fit and less than 0.90 is perceived as poor fit and Tucker-Lewis Index (TLI) greater than 0.90 indicates good fitting models. The root mean square error of approximation (RMSEA) less than 0.08 is considered acceptable fit (47, 48).

## 3 Results

### 3.1 Descriptive statistics

Table 3 presents the baseline characteristics of the analytic sample. CP was reported as non-interfering by 62.7% of participants, while 24.6% experienced low interference and 12.7% high interference pain. Regarding pain locations, 23.8% reported 1–2 pain sites, and 13.4% had pain in three or more locations. A majority of the participants were married (71.4%), presenting a diverse range of smoking statuses, with 57.5% being ex-smokers, and 60.1% classified as moderate to heavy drinkers. Lastly, the prevalence of multiple chronic conditions was nearly evenly divided among the participants, with 50.9% affirming their presence. Further information for SES indicators stratified by CP measures has shown in Appendix Tables A2, A3.

**Table 3:** Baseline characteristics of the analytic sample.

Variable	*N*	Mean/%	SD
CP variables in MIDUS 3			
Pain interference	781		
No pain	490	62.70%	
High interference pain	99	12.70%	
Low interference pain	192	24.60%	
The number of pain locations	781		
No pain	490	62.70%	
1–2	186	23.80%	
3+	105	13.40%	
Childhood covariates (collected in MIDUS 1)			
Lived with smoker growing up	781		
No	230	29.40%	
Yes	551	70.60%	
Lived with alcoholic during childhood	780		
No	611	78.30%	
Yes	169	21.70%	
Live with biological parents	781		
No	157	20.10%	
Yes	624	79.90%	
Mothers health at age 16	757		
Excellent	153	20.20%	
Very good	263	34.70%	
Good	197	26.00%	
Fair	80	10.60%	
Poor	38	5.00%	
Deceased	26	3.40%	
Fathers health at age 16	740		
Excellent	156	21.10%	
Very good	244	33.00%	
Good	182	24.60%	
Fair	81	10.90%	
Poor	38	5.10%	
Deceased	39	5.30%	
MIDUS 1 covariates			
Gender	781		
Male	351	44.90%	
Female	430	55.10%	
Ethnicity	766		
White	717	93.60%	
Non-White	49	6.40%	
Age	781	44.964	10.93
Marital status	781		
Divorced & Separated & Widowed & NM	223	28.60%	
Married	558	71.40%	
Physical activity	761	4.922	1.034
Smoking status	781		
Current smoker	101	12.90%	
Ex-smoker	449	57.50%	
non-Smoker	231	29.60%	
Drinking status	781		
Moderate + Drinker	469	60.10%	
Light Drinker	247	31.60%	
Non-drinker or rarely drink	65	8.30%	
Multimorbidity	766		
No	376	49.10%	
Yes	390	50.90%	

Appendix Table A1 presents the fit statistics for latent class model with 1–7 clusters, the 3-cluster model was considered the optimal clustering. Despite the continuous reduction in AIC and BIC, along with the progressive improvement in log-likelihood, the enhancement in the fitness of the model with 4 and 5 clusters was rather moderate. On the other hand, the 3-cluster model exhibited the best entropy, suggesting a good classification. Additionally, the 3-cluster model had an ample number of observations within each cluster and presented meaningful separation. Therefore, the 3-cluster model was adopted, and the 3 classes were labeled as ‘baseline’ as the biomarkers were in low levels of dysregulation, as ‘parasympathetic dysregulation’ and as ‘metabolic dysregulation’ (see Appendix Figure A1).

### 3.2 SEM results

Table 4 displays the estimates for continuous latent SES variables, with higher values denoting greater social disadvantage. The robust model fit indices, with a Chi-square of 188.354 at 78 degrees of freedom, a robust CFI of 0.969, a TLI of 0.952, and a RMSEA of 0.043, suggest a well-fitting model that accurately captures the covariances of SES indicators from childhood through adulthood.

**Table 4:** Confirmatory factor analysis for SES latent variables.

Latent factor					
Childhood SES		MIDUS 1 SES		MIDUS 2 SES	
Indicator	SF	Indicator	SF	Indicator	SF
Father education	0.215	Income-to-needs ratio	0.383	Income-to-needs ratio	0.239
Mother education	0.497	Education	0.259	Education	0.184
Financial level growing up	0.479	Occupation	0.161	Occupation	0.156
Father occupation	0.492	Rate current financial situation	0.483	Rate current financial situation	0.720
		Money to meet needs	0.489	Money to meet needs	0.823
		How difficult to pay monthly bills	0.552	How difficult to pay monthly bills	0.551

Chi square = 188.354; df = 78; Robust CFI = 0.969; Robust TLI = 0.952; Robust RMSEA = 0.043. SF denotes standardized factor loadings.

Table 5 shows the estimated effects of the chain of risk additive model on SES and CP. The findings suggested that previous SES exerted a subsequent direct effect on later SES. Furthermore, only the SES measured in MIDUS 2 significantly increased the odds in having high CP interference in MIDUS 3 (OR = 1.069, 95%CI = 1.006, 1.136, *p* < 0.05). Nevertheless, existing evidence did not demonstrate an additive effect of prior SES on CP in MIDUS 3 as there were no significant indirect effects of SES on CP. Table 6 shows results of the mediation analysis for AL. No indirect effects were found, indicating that AL did not mediate the association between latent SES and CP.

**Table 5:** Estimated effects of chain of risk additive model on SES and CP.

	SES->Low interference pain	SES->High interference pain	SES->1–2 pain locations	SES->3+ pain locations
Direct effects	Estimate (95%CI)	Estimate (95%CI)	Estimate (95%CI)	Estimate (95%CI)
Childhood SES->M1 SES	0.207 (0.03, 0.383)*	0.277 (0.051, 0.502)*	0.238 (0.042, 0.434)*	0.304 (0.082, 0.525)**
Childhood SES->M2 SES	−0.073 (−0.202, 0.056)	−0.044 (−0.375, 0.287)	−0.061 (−0.253, 0.131)	−0.072 (−0.391, 0.247)
M1 SES->M2 SES	0.656 (0.197, 1.114)**	1.493 (0.752, 2.233)***	0.927 (0.415, 1.438)***	1.43 (0.681, 2.178)***
	OR (95%CI)	OR (95%CI)	OR (95%CI)	OR (95%CI)
Childhood SES->Pain	0.98 (0.931, 1.031)	1.007 (0.958, 1.057)	0.991 (0.938, 1.046)	1.005 (0.956, 1.055)
M1 SES->Pain	1.02 (0.958, 1.086)	0.927 (0.813, 1.057)	0.957 (0.873, 1.05)	1.038 (0.936, 1.152)
M2 SES->Pain	0.984 (0.948, 1.021)	1.069 (1.006, 1.136)*	1.034 (0.975, 1.097)	1.002 (0.944, 1.062)
Mediation pathway	Indirect effects (SE)	Indirect effects (SE)	Indirect effects (SE)	Indirect effects (SE)
Childhood SES->M1 SES->M2 SES	0.136 (0.080)	0.414 (0.203)*	0.221 (0.114)	0.435 (0.197)*
Childhood SES->M1 SES->Pain	0.001 (0.002)	−0.003 (0.011)	−0.002 (0.004)	0.000 (0.002)
Childhood SES->M2 SES->Pain	0.004 (0.007)	−0.021 (0.021)	−0.010 (0.012)	0.012 (0.017)
Childhood SES->M1 SES->M2 SES->Pain	−0.002 (0.003)	0.028 (0.020)	0.007 (0.009)	0.001 (0.013)
M1 SES->M2 SES->Pain	−0.011 (0.012)	0.100 (0.058)	0.031 (0.033)	0.002 (0.042)
Model fitting				
Chi square	803.465	788.034	780.727	782.668
df	473	473	473	439
CFI	0.955	0.951	0.958	0.950
TLI	0.944	0.939	0.947	0.938
RMSEA	0.033	0.035	0.032	0.036

^*^p < 0.05, ^**^p < 0.01, ^***^p < 0.001. SE, denotes standard error. The bold values denote statistical significance.

**Table 6:** Chain of risk additive model on SES and CP with AL (quartile-based summation and LCA-based phenotypes).

With allostatic load (quartile-based summation)	SES->Low interference pain	SES->High interference pain	SES->1–2 pain locations	SES->3+ pain locations
Mediation pathway	Indirect effects (SE)	Indirect effects (SE)	Indirect effects (SE)	Indirect effects (SE)
Childhood SES->M1 SES->AL->Pain	0.000 (0.000)	0.000 (0.000)	0.000 (0.000)	0.000 (0.001)
Childhood SES->M1 SES->M2 SES->AL->Pain	0.000 (0.000)	0.000 (0.000)	0.000 (0.000)	0.000 (0.000)
Childhood SES->M2 SES->AL->Pain	0.000 (0.001)	0.001 (0.002)	0.000 (0.002)	0.002 (0.004)
Childhood SES->AL->Pain	0.000 (0.001)	0.000 (0.001)	0.000 (0.001)	0.001 (0.001)
M1 SES->M2 SES->AL->Pain	0.000 (0.000)	0.000 (0.000)	0.000 (0.000)	0.000 (0.000)
M1 SES->AL->Pain	0.000 (0.001)	0.000 (0.001)	0.000 (0.001)	−0.001 (0.003)
M2 SES->AL->Pain	0.000 (0.001)	0.001 (0.002)	0.000 (0.001)	0.001 (0.003)

With allostatic load (metabolic dysregulation driven)	SES->Low interference pain	SES->High interference pain	SES->1–2 pain locations	SES->3+ pain locations
Mediation pathway	Indirect effects (SE)	Indirect effects (SE)	Indirect effects (SE)	Indirect effects (SE)
Childhood SES->M1 SES->AL->Pain	0.000 (0.000)	0.000 (0.000)	0.000 (0.000)	0.000 (0.000)
Childhood SES->M1 SES->M2 SES->AL->Pain	0.000 (0.000)	0.000 (0.000)	0.000 (0.000)	0.000 (0.000)
Childhood SES->M2 SES->AL->Pain	−0.002 (0.003)	0.001 (0.001)	−0.001 (0.002)	0.001 (0.002)
Childhood SES->AL->Pain	0.001 (0.002)	0.000 (0.001)	0.001 (0.002)	0.000 (0.001)
M1 SES->M2 SES->AL->Pain	0.000 (0.000)	0.000 (0.000)	0.000 (0.000)	0.000 (0.000)
M1 SES->AL->Pain	0.000 (0.002)	0.000 (0.001)	0.000 (0.001)	0.000 (0.001)
M2 SES->AL->Pain	−0.002 (0.003)	0.001 (0.001)	−0.001 (0.002)	0.001 (0.002)

With allostatic load (parasympathetic dysregulation driven)	SES->Low interference pain	SES->High interference pain	SES->1–2 pain locations	SES->3+ pain locations
Mediation pathway	Indirect effects (SE)	Indirect effects (SE)	Indirect effects (SE)	Indirect effects (SE)
Childhood SES->M1 SES->AL->Pain	0.000 (0.000)	0.000 (0.000)	0.000 (0.000)	0.000 (0.001)
Childhood SES->M1 SES->M2 SES->AL->Pain	0.000 (0.000)	0.000 (0.000)	0.000 (0.000)	0.000 (0.000)
Childhood SES->M2 SES->AL->Pain	0.000 (0.000)	0.000 (0.002)	0.000 (0.002)	0.001 (0.003)
Childhood SES->AL->Pain	0.000 (0.002)	0.000 (0.000)	0.000 (0.002)	0.000 (0.001)
M1 SES->M2 SES->AL->Pain	0.000 (0.000)	0.000 (0.000)	0.000 (0.000)	0.000 (0.000)
M1 SES->AL->Pain	0.000 (0.001)	0.000 (0.000)	0.000 (0.001)	−0.001 (0.003)
M2 SES->AL->Pain	0.000 (0.000)	0.000 (0.001)	0.000 (0.002)	0.001 (0.002)

^*^p < 0.05, ^**^p < 0.01, ^***^p < 0.001. SE, denotes standard error.

The study subsequently conducted sensitivity analyses to assess the robustness of the significant direct effects of the SES latent variable in MIDUS 2 on CP in MIDUS 3. Firstly, to mitigate the impact of retirement on SES and CP, as well as the influence of escalating multimorbidity in later life on CP, supplementary SEM for participants aged below 65 was conducted. The results continued to be significant. Additionally, respondents who reported an absence of a female or male head in their family for most of their childhood were excluded and the findings remained significant.

## 4 Discussion

Using MIDUS data, this study investigated the prospective association between life course SES and CP outcomes. Our findings demonstrated the SES latent variable in MIDUS 2 increased odds of having high-interference CP in MIDUS 3. However, this study did not demonstrate an additive SES effect on the odds of having CP. Specifically, both childhood SES and SES in MIDUS 1 did not impact CP, and no association was found regarding their indirect effects. Also, the effects of SES on CP were not mediated by AL.

During MIDUS 2 survey, the average age of participants was 54 years (SD = 10.91), with the latent SES variable in this age group being associated with increased pain interference odds in MIDUS 3. At the time of MIDUS 3 survey, the average follow-up age was 63 years (SD = 10.912). Previous studies have identified a significant association between home ownership and CWP in a similar age group (12). Therefore, the current study may partly support the hypothesis that midlife SES constitutes a sensitive period influencing odds in having CP in later life. Nonetheless, this study did not observe a prospective association between SES in MIDUS 1 (mean age 45 years, SD = 10.93) and the odds of CP in MIDUS 3. On one hand, although previous research found that financial hardships at the age of 43 are linked to CWP at age 68 (12), thereby elucidating the distinct impact of single SES indicators on CP, this approach did not adequately capture the complex interplay between these factors. Moreover, it fails to reflect the multidimensional nature of SES (8).

On the other hand, the different results compared to birth cohort studies might be attributed not only to the age heterogeneity among MIDUS 1 respondents but also to the fact that a two-decade time span potentially diminishes the influence of SES. Shorter data collection intervals could heighten the sensitivity of CP to SES effects (13). Therefore, the interpretation of the age-sensitive period warrants caution, as a portion of the significant effect observed in MIDUS 2 SES may be ascribed to proximal effects. Comparable recency effects have been documented in life course SES studies concerning other chronic diseases. For example, a study using the Wisconsin Longitudinal Study (WLS) found that proximal SES, directly affected mortality in later life (49). Another study used a 27-year prospective cohort from northern Sweden and found only recent SES was predictive of AL in the total sample and in the male sample (50).

In the sensitivity analysis, this study found no association between SES among respondents aged over 65 years at MIDUS 2 and the odds of having CP at MIDUS 3, aligning with findings from prior research (12). With advancing age, the predominance of aging and associated geriatric diseases emerges as key pathogenic mechanisms in CP among older people (51) and the explanatory power of SES in accounting for variations in reporting CP diminishes. Research that collects CP information in younger cohorts can offer more profound insights into the relationship between SES and CP. On the other hand, research suggests that more severe cases of CP are linked with shorter follow-up periods due to decease attrition (52). Therefore, mortality related to CP potentially underestimates the severity of CP as individuals age. Future research could be enhanced by employing longitudinal assessments of CP and stratifying participants based on their follow-up duration until attrition. Such an approach would help ascertain whether life course SES is related to widening disparities in the odds of reporting CP.

Contrary to the findings of this study, earlier research has suggested that experiencing financial hardship at the ages of 43, and between 60 and 64, can have cumulative effects (12). However, this significance was largely due to the financial hardship experienced at age 43. This summation approach might inadvertently overstate the relatively minor association of financial hardship with the 60–64 age group. Also, financial hardship exhibited a more pronounced effect at the age of 43 than its cumulative impact observed between the ages of 43 and 60–64. Therefore, the cumulative effect remains unclear. Utilizing path analysis, this study was able to separately quantify the effects of subsequent exposures (53), thus overcoming the strict assumption that every increase in score at different times has an equal impact on CP.

Another result inconsistent with previous studies is that this study did not find an association with the number of CP sites. CWP measured in previous studies was defined according to the criteria of the American College of Rheumatology (ACR), which included pain in contralateral body quadrants and the axial skeleton persisting for more than three months (13). The definition of CWP according to ACR criteria may signify a more severe condition potentially resulting in a stronger correlation.

Although prior studies have highlighted the potential mediating role of AL, this research did not demonstrate a mediating effect of AL in the prospective association between SES and CP. Similarly, a cross-sectional study in the United States found no mediating effect of AL between SES and CP (30). However, their definition of pain accorded with acute rather than chronic conditions, potentially diminishing mediation effects of AL. Acute pain often results from specific diseases or injuries (54) and may not involve the same chronic stress-related pathways as CP. Furthermore, the CP measures utilized in this study assessed a general CP profile of the participants, wherein specific CP subtypes may not be associated with the dysregulation of the chronic stress response (55). Therefore, a general measure of CP could obscure the potential correlation between AL and CP specific to chronic stress (56). Future research may benefit from exploring CP subtypes and alternative intermediate mechanisms, including psychosocial and health behavioral factors (8).

This study has following additional advantages. Firstly, by integrating both primary and secondary biological indicators of chronic stress response to construct AL, it may enhance the measurement’s validity. This study not only employed the classic summative method for operationalizing AL but also utilized LCA to capture the interrelations among AL biomarkers. By integrating AL phenotypes into the mediation analysis, the study may provide a comprehensive assessment. Finally, the prospective nature of this study allows for the inclusion of early confounders, thereby minimizing potential confounding effects and establishing a temporal sequence in the relationship between SES and CP.

There are limitations in the present study. Although the demographic and health characteristics of the biomarker sample closely align with those of the national survey sample (57), the analytic sample demonstrates an underrepresentation of ethnic minorities. Racial disparities in health outcomes are a profound concern in the United States (58). The increasing disparity in the prevalence and treatment of CP among ethnic minorities, driven by structural factors such as discrimination and the chronic stress of socioeconomic disadvantage, calls for attention (59). Thus, future studies can prioritize the inclusion of minority ethnic groups to address this gap. Also, the association between SES and CP may be influenced by attrition bias, as participants with more SES disadvantages at baseline were more likely to drop out. This could lead to an underestimation of the true impact of SES on CP outcomes (see Appendix Table B1). Furthermore, childhood indicators were measured retrospectively, which are subject to recall bias. However, the impact of this bias might be minimal. Studies validating the concordance of childhood SES indicators in MIDUS, using sibling and twin samples, have shown that recall measures were generally reliable (60). Nonetheless, employing prospective indicators for childhood conditions is recommended for future research. While SEM was employed to assess SES, certain variables showed variability in their factor loadings. This variability underlines the potential to refine SES indicators within the model, aiming for more accurate and representative measures in future research. Finally, employing repeated measures of SES and CP might more accurately track the dynamic interplay between these variables.

## 5 Conclusion

In a community-dwelling sample of US adults, proximal SES was associated with high-interference CP. The finding emphasizes equitable health resources distribution and policies aimed at reducing socioeconomic disparities, which in turn could alleviate the burden of CP in lower SES groups. The lack of indirect effects of AL highlights the complexity of CP’s etiology and necessitates a multifaceted approach to research and treatment. Future research could explore other biological, psychological, or social factors that might mediate the relationship between proximal SES and CP to improve the efficacy of interventions for individuals suffering from CP.

## Data availability statement

The original contributions presented in the study are included in the article/Supplementary material, further inquiries can be directed to the corresponding author.

## Ethics statement

The studies involving humans were approved by Education and Social/Behavioral Sciences and the Health Sciences IRBs at the University of Wisconsin-Madison. The studies were conducted in accordance with the local legislation and institutional requirements. The participants provided their written informed consent to participate in this study.

## Author contributions

YL: Conceptualization, Data curation, Formal analysis, Methodology, Software, Visualization, Writing – original draft, Writing – review & editing.

## Footnotes

^1^Available at: http://midus.wisc.edu/, Accessed on October 2, 2022.

## References

[1] Treede R-D Rief W Barke A Aziz Q Bennett MI Benoliel R. Chronic pain as a symptom or a disease: the IASP classification of chronic pain for the international classification of diseases (ICD-11). Pain. (2019) 160:19–27. doi: 10.1097/j.pain.0000000000001384 30586067

[2] Yong RJ Mullins PM Bhattacharyya N. Prevalence of chronic pain among adults in the United States. Pain. (2022) 163:e328–32. doi: 10.1097/j.pain.0000000000002291 33990113

[3] Zajacova A Grol-Prokopczyk H Zimmer Z. Pain trends among American adults, 2002–2018: patterns, disparities, and correlates. Demography. (2021) 58:711–38. doi: 10.1215/00703370-8977691, PMID: 33834222 PMC8035485

[4] Zimmer Z Zajacova A. Persistent, consistent, and extensive: The trend of increasing pain prevalence in older Americans. J Gerontol. (2018) 75:436–47. doi: 10.1093/geronb/gbx162, PMID: 29579314 PMC12098924

[5] Zajacova A Grol-Prokopczyk H Zimmer Z. Sociology of chronic pain. J Health Soc Behav. (2021) 62:302–17. doi: 10.1177/00221465211025962, PMID: 34283649 PMC8956223

[6] Link BG Phelan J. Social conditions as fundamental causes of disease. J Health Soc Behav. (1995) 35:80. doi: 10.2307/2626958 7560851

[7] Phelan JC Link BG Tehranifar P. Social conditions as fundamental causes of health inequalities: theory, evidence, and policy implications. J Health Soc Behav. (2010) 51:S28–40. doi: 10.1177/0022146510383498, PMID: 20943581

[8] Khalatbari-Soltani S Blyth FM. Socioeconomic position and pain: a topical review. Pain. (2022) 163:1855–61. doi: 10.1097/j.pain.0000000000002634, PMID: 35297800

[9] Ben-Shlomo Y Kuh D. A life course approach to chronic disease epidemiology: conceptual models, empirical challenges and interdisciplinary perspectives. Int J Epidemiol. (2002) 31:285–93. doi: 10.1093/ije/31.2.285, PMID: 11980781

[10] Nelson CA Gabard-Durnam LJ. Early adversity and critical periods: neurodevelopmental consequences of violating the expectable environment. Trends Neurosci. (2020) 43:133–43. doi: 10.1016/j.tins.2020.01.002, PMID: 32101708 PMC8092448

[11] Ahrens W Pigeot I. Life course epidemiology In: Handbook of epidemiology. Eds. Yoav Ben-Shlomo, Gita Mishra and Diana Kuh. New York, NY: Springer (2014).

[12] Jay MA Bendayan R Cooper R Muthuri SG. Lifetime socioeconomic circumstances and chronic pain in later adulthood: findings from a British birth cohort study. BMJ Open. (2019) 9:e024250. doi: 10.1136/bmjopen-2018-024250, PMID: 30850405 PMC6429846

[13] Macfarlane GJ Norrie G Atherton K Power C Jones GT. The influence of socioeconomic status on the reporting of regional and widespread musculoskeletal pain: results from the 1958 British birth cohort study. Ann Rheum Dis. (2009) 68:1591–5. doi: 10.1136/ard.2008.093088, PMID: 18952642

[14] Celeste RK Fritzell J. Do socioeconomic inequalities in pain, psychological distress and oral health increase or decrease over the life course? Evidence from Sweden over 43 years of follow-up. J Epidemiol Community Health. (2018) 72:160–7. doi: 10.1136/jech-2017-209123, PMID: 29175868 PMC5800356

[15] Goosby BJ. Early life course pathways of adult depression and chronic pain. J Health Soc Behav. (2013) 54:75–91. doi: 10.1177/0022146512475089, PMID: 23426854 PMC3733784

[16] Lacey RJ Belcher J Croft PR. Does life course socio-economic position influence chronic disabling pain in older adults? A general population study. European J Public Health. (2013) 23:534–40. doi: 10.1093/eurpub/cks056, PMID: 22874735 PMC3719471

[17] Hoffmann R Kröger H Pakpahan E. Pathways between socioeconomic status and health: does health selection or social causation dominate in Europe? Adv Life Course Res. (2018) 36:23–36. doi: 10.1016/j.alcr.2018.02.002

[18] Adler NE Boyce T Chesney MA Cohen S Folkman S Kahn RL. Socioeconomic status and health. The challenge of the gradient. Am Psychol. (1994) 49:15–24. doi: 10.1037//0003-066x.49.1.15 8122813

[19] Rios R Zautra AJ. Socioeconomic disparities in pain: The role of economic hardship and daily financial worry. Health Psychol. (2011) 30:58–66. doi: 10.1037/a0022025, PMID: 21299295 PMC3077089

[20] Juster R-P McEwen BS Lupien SJ. Allostatic load biomarkers of chronic stress and impact on health and cognition. Neurosci Biobehav Rev. (2010) 35:2–16. doi: 10.1016/j.neubiorev.2009.10.002, PMID: 19822172

[21] McEwen BS. Stress, adaptation, and disease: Allostasis and allostatic load. Ann N Y Acad Sci. (1998) 840:33–44. doi: 10.1111/j.1749-6632.1998.tb09546.x 9629234

[22] Herman JP McKlveen JM Ghosal S Kopp B Wulsin A Makinson R. Regulation of the hypothalamic-pituitary-adrenocortical stress response. Compr Physiol. (2016) 6:603–21. doi: 10.1002/cphy.c150015, PMID: 27065163 PMC4867107

[23] Dowd JB Simanek AM Aiello AE. Socio-economic status, cortisol and allostatic load: a review of the literature. Int J Epidemiol. (2009) 38:1297–309. doi: 10.1093/ije/dyp277, PMID: 19720725 PMC2755130

[24] Johnson SC Cavallaro FL Leon DA. A systematic review of allostatic load in relation to socioeconomic position: poor fidelity and major inconsistencies in biomarkers employed. Soc Sci Med. (2017) 192:66–73. doi: 10.1016/j.socscimed.2017.09.025, PMID: 28963986

[25] Abdallah CG Geha P. Chronic pain and chronic stress: two sides of the same coin? Chronic Stress. (2017) 1:247054701770476. doi: 10.1177/2470547017704763, PMID: 28795169 PMC5546756

[26] Woda A Picard P Dutheil F. Dysfunctional stress responses in chronic pain. Psychoneuroendocrinology. (2016) 71:127–35. doi: 10.1016/j.psyneuen.2016.05.017 27262345

[27] Borsook D Maleki N Becerra L McEwen B. Understanding migraine through the Lens of maladaptive stress responses: a model disease of allostatic load. Neuron. (2012) 73:219–34. doi: 10.1016/j.neuron.2012.01.001, PMID: 22284178

[28] Sibille KT McBeth J Smith D Wilkie R. Allostatic load and pain severity in older adults: results from the English longitudinal study of ageing. Exp Gerontol. (2017) 88:51–8. doi: 10.1016/j.exger.2016.12.013, PMID: 27988258 PMC5326483

[29] Sibille KT Steingrímsdóttir ÓA Fillingim RB Stubhaug A Schirmer H Chen H. Investigating the burden of chronic pain: an inflammatory and metabolic composite. Pain Res Manag. (2016) 2016:1–11. doi: 10.1155/2016/7657329, PMID: 27445627 PMC4909918

[30] Slade GD Sanders AE By K. Role of allostatic load in sociodemographic patterns of pain prevalence in the U.S. Pain forum. (2012) 13:666–75. doi: 10.1016/j.jpain.2012.04.003, PMID: 22677453 PMC3652569

[31] Carbone JT Clift J Alexander N. Measuring allostatic load: approaches and limitations to algorithm creation. J Psychosom Res. (2022) 163:111050. doi: 10.1016/j.jpsychores.2022.111050, PMID: 36228435

[32] Guerriero F Reid MC. Pain and healthy aging In: Coll PP, editor. Healthy aging: A complete guide to clinical management. Cham: Springer International Publishing (2019). 305–12.

[33] Von Korff M Dworkin SF Le Resche L. Graded chronic pain status: an epidemiologic evaluation. Pain. (1990) 40:279–91. doi: 10.1016/0304-3959(90)91125-3 2326094

[34] Cleeland CS Ryan KM. Pain assessment: global use of the brief pain inventory. Ann Acad Med Singap. (1994) 23:129–38. PMID: 8080219

[35] Jensen MP. Measuring pain interference In: The pain stethoscope: A Clinician’s guide to measuring pain. Ed. Tamsin Curtis. Tarporley: Springer Healthcare Ltd. (2011). 23–7.

[36] Li R Kreher DA Jusko TA Chapman BP Bonham AD Seplaki CL. Prospective association between dysmenorrhea and chronic pain development in community-dwelling women. J Pain. (2021) 22:1084–96. doi: 10.1016/j.jpain.2021.03.139, PMID: 33762206

[37] Gruenewald TL Karlamangla AS Hu P Stein-Merkin S Crandall C Koretz B. History of socioeconomic disadvantage and allostatic load in later life. Soc Sci Med. (2012) 74:75–83. doi: 10.1016/j.socscimed.2011.09.037, PMID: 22115943 PMC3264490

[38] Hastings WJ Almeida DM Shalev I. Conceptual and analytical overlap between allostatic load and systemic biological aging measures: analyses from the National Survey of midlife development in the United States. J Gerontol. (2022) 77:1179–88. doi: 10.1093/gerona/glab187 PMC9159656 34180993

[39] Karlamangla AS Miller-Martinez D Lachman ME Tun PA Koretz BK Seeman TE. Biological correlates of adult cognition: midlife in the United States (MIDUS). Neurobiol Aging. (2014) 35:387–94. doi: 10.1016/j.neurobiolaging.2013.07.028, PMID: 24011541 PMC3830604

[40] McEwen BS Seeman T. Protective and damaging effects of mediators of stress: elaborating and testing the concepts of Allostasis and allostatic load. Ann N Y Acad Sci. (1999) 896:30–47. doi: 10.1111/j.1749-6632.1999.tb08103.x, PMID: 10681886

[41] Weller BE Bowen NK Faubert SJ. Latent class analysis: a guide to best practice. J Black Psychol. (2020) 46:287–311. doi: 10.1177/0095798420930932

[42] Sinha P Calfee CS Delucchi KL. Practitioner’s guide to latent class analysis: methodological considerations and common pitfalls. Crit Care Med. (2021) 49:e63–79. doi: 10.1097/CCM.0000000000004710, PMID: 33165028 PMC7746621

[43] Glover LM Martin CL Green-Howard A Adatorwovor R Loehr L Staley-Salil B. Cumulative socioeconomic status and incident type 2 diabetes among African American adults from the Jackson heart study. SSM Populat Health. (2023) 22:101389. doi: 10.1016/j.ssmph.2023.101389 PMC10165449 37168250

[44] Surachman A Wardecker B Chow S-M Almeida D. Life course socioeconomic status, daily stressors, and daily well-being: examining chain of risk models. J Gerontol. (2019) 74:126–35. doi: 10.1093/geronb/gby014, PMID: 29669043 PMC6294233

[45] Ryff C.D. Seeman T. Weinstein M. (2018). ICPSR 29282 midlife in the United States (MIDUS 2): Biomarker project, 2004–2009 scales and composite variables. Inter-university Consortium for Political and Social Research [distributor].

[46] Enders CK Bandalos DL. The relative performance of full information maximum likelihood estimation for missing data in structural equation models. Struct Equ Model Multidiscip J. (2001) 8:430–57. doi: 10.1207/S15328007SEM0803_5

[47] Hu L Bentler PM. Cutoff criteria for fit indexes in covariance structure analysis: conventional criteria versus new alternatives. Struct Equ Model Multidiscip J. (1999) 6:1–55. doi: 10.1080/10705519909540118

[48] Yuan K-H Chan W Marcoulides GA Bentler PM. Assessing structural equation models by equivalence testing with adjusted fit indexes. Struct Equ Model Multidiscip J. (2016) 23:319–30. doi: 10.1080/10705511.2015.1065414

[49] Pudrovska T Anikputa B. Early-life socioeconomic status and mortality in later life: an integration of four life-course mechanisms. J Gerontol. (2014) 69:451–60. doi: 10.1093/geronb/gbt122, PMID: 24496607 PMC3983914

[50] Gustafsson PE Janlert U Theorell T Westerlund H Hammarstrom A. Socioeconomic status over the life course and allostatic load in adulthood: results from the northern Swedish cohort. J Epidemiol Community Health. (2011) 65:986–92. doi: 10.1136/jech.2010.108332, PMID: 20974835

[51] Domenichiello AF Ramsden CE. The silent epidemic of chronic pain in older adults. Prog Neuro-Psychopharmacol Biol Psychiatry. (2019) 93:284–90. doi: 10.1016/j.pnpbp.2019.04.006, PMID: 31004724 PMC6538291

[52] Grol-Prokopczyk H. Sociodemographic disparities in chronic pain, based on 12-year longitudinal data. Pain. (2017) 158:313–22. doi: 10.1097/j.pain.0000000000000762, PMID: 28092650 PMC5242384

[53] Green MJ Popham F. Life course models: improving interpretation by consideration of total effects. Int J Epidemiol. (2017) 46:dyw329–dyw1062. doi: 10.1093/ije/dyw329, PMID: 28031311 PMC5837734

[54] Grichnik KP Ferrante FM. The difference between acute and chronic pain. Mt Sinai J Med. (1991) 58:217–20. PMID: 1875958

[55] Cohen SP Vase L Hooten WM. Chronic pain: an update on burden, best practices, and new advances. Lancet. (2021) 397:2082–97. doi: 10.1016/S0140-6736(21)00393-7, PMID: 34062143

[56] Nicholas M Vlaeyen JWS Rief W Barke A Aziz Q Benoliel R. The IASP classification of chronic pain for ICD-11: chronic primary pain. Pain. (2019) 160:28–37. doi: 10.1097/j.pain.0000000000001390, PMID: 30586068

[57] Dienberg Love G Seeman TE Weinstein M Ryff CD. Bioindicators in the MIDUS National Study: protocol, measures, sample, and comparative context. J Aging Health. (2010) 22:1059–80. doi: 10.1177/0898264310374355, PMID: 20876364 PMC2972372

[58] Clouston SAP Link BG. A retrospective on fundamental cause theory: state of the literature and goals for the future. Annu Rev Sociol. (2021) 47:131–56. doi: 10.1146/annurev-soc-090320-094912, PMID: 34949900 PMC8691558

[59] Maly A Vallerand AH. Neighborhood, socioeconomic, and racial influence on chronic pain. Pain Manag Nurs. (2018) 19:14–22. doi: 10.1016/j.pmn.2017.11.004, PMID: 29422123 PMC8895435

[60] Ward MM. Concordance of sibling’s recall of measures of childhood socioeconomic position. BMC Med Res Methodol. (2011) 11:147. doi: 10.1186/1471-2288-11-147 22044489 PMC3261820

