# Supplementary material for: Life course socioeconomic status, chronic pain, and the mediating role of allostatic load: findings from the midlife in the United States
Source: Front Public Health. 2024 Mar 18;12:1365105. doi: 10.3389/fpubh.2024.1365105 (PMC10982432; doi:10.3389/fpubh.2024.1365105)
Supplement: Supplementary file 1 [file Data_Sheet_1.docx]

**Table A.1 Fitting statistics for latent class modeling of allostatic load driven pattern.**

| **LCA** | **llik** | **AIC** | **BIC** | **SABIC** | **Entropy** | **Class 1** | **Class 2** | **Class 3** | **Class 4** | **Class 5** | **Class 6** | **Class 7** |
| --- | --- | --- | --- | --- | --- | --- | --- | --- | --- | --- | --- | --- |
| 1 | -11670.67 | 23395.33 | 23521.17 | 23341.33 | NaN | 100% |  |  |  |  |  |  |
| 2 | -11067.14 | 22244.28 | 22500.61 | 22134.28 | 0.8460038 | 62% | 38% |  |  |  |  |  |
| 3 | -10813.98 | 21793.96 | 22180.79 | 21627.96 | 0.8553862 | 25% | 51% | 24% |  |  |  |  |
| 4 | -10704.2 | 21630.4 | 22147.73 | 21408.4 | 0.8450053 | 22% | 47% | 17% | 14% |  |  |  |
| 5 | -10606.58 | 21491.16 | 22138.98 | 21213.16 | 0.8398492 | 14% | 11% | 15% | 17% | 43% |  |  |
| 6 | -10528.3 | 21390.6 | 22168.91 | 21056.6 | NaN | 13% | 17% | 13% | 11% | 27% | 20% |  |
| 7 | -10468.26 | 21326.53 | 22235.34 | 20936.53 | NaN | 11% | 19% | 29% | 7% | 10% | 13% | 12% |

**Figure A.1 Allostatic load phenotypes**


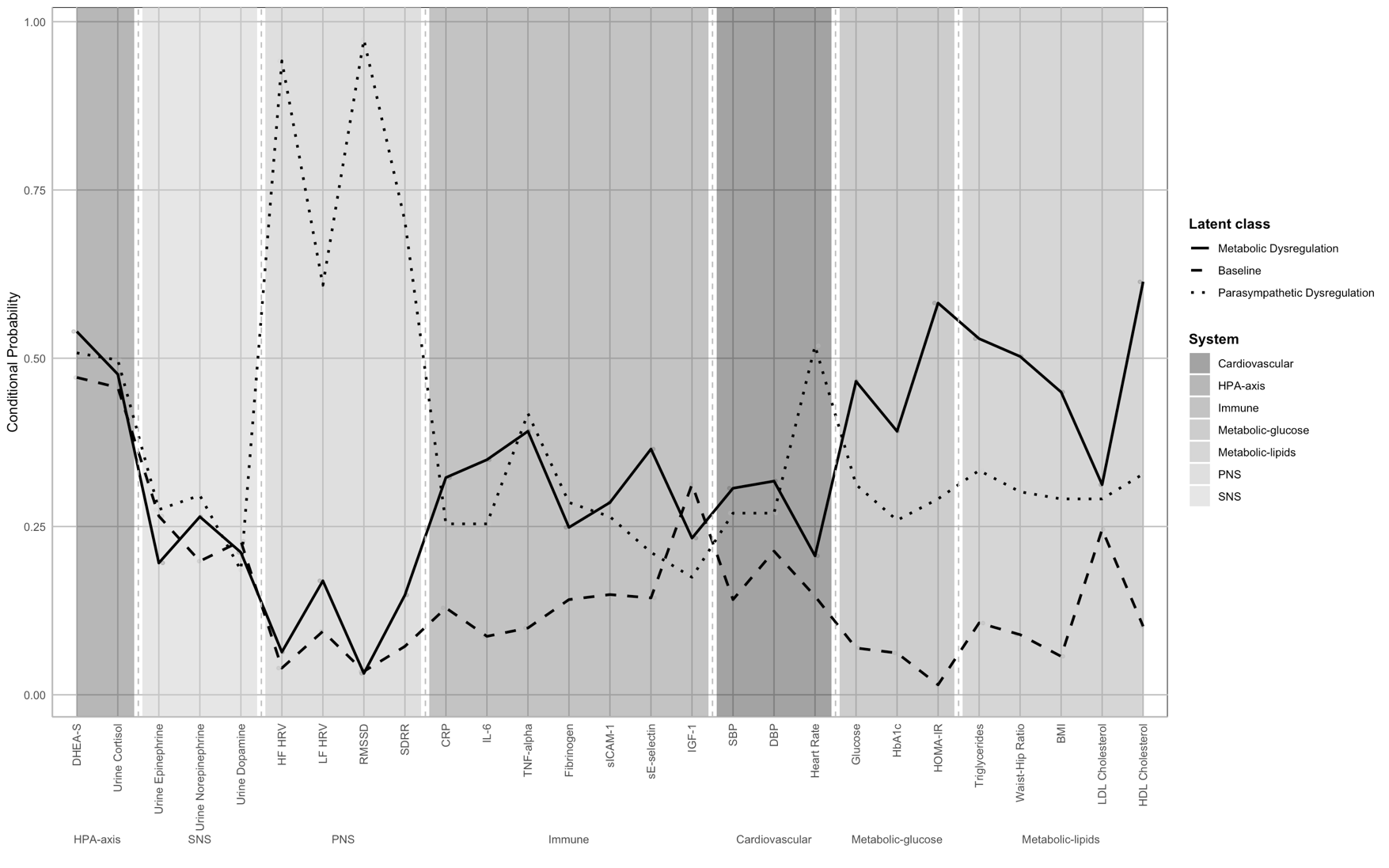


**Table A.2 Distribution of SES indicators stratified by CP interference**

|  | **No pain** | | | **High interference pain** | | | **Low interference pain** | | |  |
| --- | --- | --- | --- | --- | --- | --- | --- | --- | --- | --- |
|  | **N** | **Mean / %** | **Sd** | **N** | **Mean / %** | **Sd** | **N** | **Mean / %** | **Sd** | **Test** |
| **Childhood SES indicators** |  |  |  |  |  |  |  |  |  |  |
| Fathers education | 454 |  |  | 91 |  |  | 177 |  |  | X2=9.301^*^ |
| less than high school | 153 | 33.70% |  | 40 | 44.00% |  | 58 | 32.80% |  |  |
| high school/GED/some college | 210 | 46.30% |  | 27 | 29.70% |  | 84 | 47.50% |  |  |
| bachelor's degree or more | 91 | 20.00% |  | 24 | 26.40% |  | 35 | 19.80% |  |  |
| Mother education | 477 |  |  | 96 |  |  | 187 |  |  | X2=2.543 |
| less than high school | 123 | 25.80% |  | 28 | 29.20% |  | 54 | 28.90% |  |  |
| high school/GED/some college | 276 | 57.90% |  | 57 | 59.40% |  | 101 | 54.00% |  |  |
| bachelor's degree or more | 78 | 16.40% |  | 11 | 11.50% |  | 32 | 17.10% |  |  |
| Financial level growing up | 482 |  |  | 95 |  |  | 188 |  |  | X2=16.412 |
| A lot better off | 14 | 2.90% |  | 3 | 3.20% |  | 3 | 1.60% |  |  |
| Somewhat better off | 65 | 13.50% |  | 11 | 11.60% |  | 23 | 12.20% |  |  |
| A little better off | 68 | 14.10% |  | 11 | 11.60% |  | 40 | 21.30% |  |  |
| Same as average family | 207 | 42.90% |  | 33 | 34.70% |  | 65 | 34.60% |  |  |
| A little worse off | 85 | 17.60% |  | 22 | 23.20% |  | 37 | 19.70% |  |  |
| Somewhat worse off | 31 | 6.40% |  | 11 | 11.60% |  | 17 | 9.00% |  |  |
| A lot worse off | 12 | 2.50% |  | 4 | 4.20% |  | 3 | 1.60% |  |  |
| Father's occupation |  |  |  |  |  |  |  |  |  |  |
| Managerial And Professional Specialty Occupations | 451 |  |  | 91 |  |  | 177 |  |  | X2=9.12 |
| Technical, Sales, And Administrative Support Occupations | 134 | 29.70% |  | 26 | 28.60% |  | 53 | 29.90% |  |  |
| Service Occupations | 74 | 16.40% |  | 14 | 15.40% |  | 37 | 20.90% |  |  |
| Farming, Forestry, And Fishing Occupations | 19 | 4.20% |  | 2 | 2.20% |  | 6 | 3.40% |  |  |
| Precision Production, Craft, And Repair Occupations | 74 | 16.40% |  | 9 | 9.90% |  | 22 | 12.40% |  |  |
| Operators, Fabricators, And Laborers | 75 | 16.60% |  | 20 | 22.00% |  | 29 | 16.40% |  |  |
| Experienced Unemployed Not Classified By Occupations | 62 | 13.70% |  | 17 | 18.70% |  | 27 | 15.30% |  |  |
| **M1 SES indicators** | 13 | 2.90% |  | 3 | 3.30% |  | 3 | 1.70% |  |  |
| Rate current financial situation | 481 | 6.428 | 2.14 | 93 | 5.839 | 2.133 | 187 | 6.251 | 2.104 | F=3.088^**^ |
| Money to meet needs | 482 |  |  | 95 |  |  | 188 |  |  | X2=13.82^***^ |
| More than enough money | 110 | 22.80% |  | 14 | 14.70% |  | 44 | 23.40% |  |  |
| Just enough money | 274 | 56.80% |  | 46 | 48.40% |  | 106 | 56.40% |  |  |
| Not enough money | 98 | 20.30% |  | 35 | 36.80% |  | 38 | 20.20% |  |  |
| How difficult to pay monthly bills | 483 |  |  | 95 |  |  | 187 |  |  | X2=20.274^***^ |
| Not at all difficult | 173 | 35.80% |  | 14 | 14.70% |  | 58 | 31.00% |  |  |
| Not very difficult | 173 | 35.80% |  | 40 | 42.10% |  | 75 | 40.10% |  |  |
| Somewhat difficult | 115 | 23.80% |  | 35 | 36.80% |  | 41 | 21.90% |  |  |
| Very difficult | 22 | 4.60% |  | 6 | 6.30% |  | 13 | 7.00% |  |  |
| Education | 490 |  |  | 99 |  |  | 191 |  |  | X2=18.38^***^ |
| less than high school | 7 | 1.40% |  | 9 | 9.10% |  | 7 | 3.70% |  |  |
| high school/GED/some college | 251 | 51.20% |  | 52 | 52.50% |  | 99 | 51.80% |  |  |
| bachelor's degree or more | 232 | 47.30% |  | 38 | 38.40% |  | 85 | 44.50% |  |  |
| Income-to-needs ratio | 475 |  |  | 95 |  |  | 186 |  |  | X2=14.382^*^ |
| Affluent | 371 | 78.10% |  | 59 | 62.10% |  | 134 | 72.00% |  |  |
| Adequate-income | 69 | 14.50% |  | 23 | 24.20% |  | 36 | 19.40% |  |  |
| Low-income | 22 | 4.60% |  | 9 | 9.50% |  | 8 | 4.30% |  |  |
| Poor | 4 | 0.80% |  | 2 | 2.10% |  | 2 | 1.10% |  |  |
| Extreme poverty | 9 | 1.90% |  | 2 | 2.10% |  | 6 | 3.20% |  |  |
| Occupation | 480 |  |  | 90 |  |  | 186 |  |  | X2=16.106 |
| Managerial And Professional Specialty Occupations | 220 | 45.80% |  | 41 | 45.60% |  | 88 | 47.30% |  |  |
| Technical, Sales, And Administrative Support Occupations | 133 | 27.70% |  | 17 | 18.90% |  | 51 | 27.40% |  |  |
| Service Occupations | 42 | 8.80% |  | 13 | 14.40% |  | 9 | 4.80% |  |  |
| Farming, Forestry, And Fishing Occupations | 13 | 2.70% |  | 2 | 2.20% |  | 2 | 1.10% |  |  |
| Precision Production, Craft, And Repair Occupations | 33 | 6.90% |  | 8 | 8.90% |  | 15 | 8.10% |  |  |
| Operators, Fabricators, And Laborers | 29 | 6.00% |  | 6 | 6.70% |  | 11 | 5.90% |  |  |
| Experienced Unemployed Not Classified By Occupations | 10 | 2.10% |  | 3 | 3.30% |  | 10 | 5.40% |  |  |
| **M2 SES indicators** |  |  |  |  |  |  |  |  |  |  |
| Rate current financial situation | 485 | 6.713 | 2.028 | 98 | 5.612 | 2.519 | 192 | 6.609 | 2.061 | F=11.271^***^ |
| Money to meet needs | 489 |  |  | 99 |  |  | 192 |  |  | X2=26.643^***^ |
| More than enough money | 170 | 34.80% |  | 22 | 22.20% |  | 64 | 33.30% |  |  |
| Just enough money | 242 | 49.50% |  | 42 | 42.40% |  | 103 | 53.60% |  |  |
| Not enough money | 77 | 15.70% |  | 35 | 35.40% |  | 25 | 13.00% |  |  |
| How difficult to pay monthly bills | 489 |  |  | 99 |  |  | 192 |  |  | X2=51.015^***^ |
| Not at all difficult | 223 | 45.60% |  | 21 | 21.20% |  | 73 | 38.00% |  |  |
| Not very difficult | 161 | 32.90% |  | 29 | 29.30% |  | 75 | 39.10% |  |  |
| Somewhat difficult | 90 | 18.40% |  | 35 | 35.40% |  | 39 | 20.30% |  |  |
| Very difficult | 15 | 3.10% |  | 14 | 14.10% |  | 5 | 2.60% |  |  |
| Education | 489 |  |  | 99 |  |  | 192 |  |  | X2=24.09^***^ |
| less than high school | 4 | 0.80% |  | 9 | 9.10% |  | 8 | 4.20% |  |  |
| high school/GED/some college | 236 | 48.30% |  | 47 | 47.50% |  | 93 | 48.40% |  |  |
| bachelor's degree or more | 249 | 50.90% |  | 43 | 43.40% |  | 91 | 47.40% |  |  |
| Income-to-needs ratio | 483 |  |  | 97 |  |  | 187 |  |  | X2=14.769^*^ |
| Affluent | 292 | 60.50% |  | 49 | 50.50% |  | 96 | 51.30% |  |  |
| Adequate-income | 129 | 26.70% |  | 29 | 29.90% |  | 53 | 28.30% |  |  |
| Low-income | 34 | 7.00% |  | 6 | 6.20% |  | 19 | 10.20% |  |  |
| Poor | 8 | 1.70% |  | 6 | 6.20% |  | 6 | 3.20% |  |  |
| Extreme poverty | 20 | 4.10% |  | 7 | 7.20% |  | 13 | 7.00% |  |  |
| Occupation | 471 |  |  | 88 |  |  | 184 |  |  | X2=39.053^***^ |
| Managerial And Professional Specialty Occupations | 223 | 47.30% |  | 40 | 45.50% |  | 97 | 52.70% |  |  |
| Technical, Sales, And Administrative Support Occupations | 128 | 27.20% |  | 10 | 11.40% |  | 42 | 22.80% |  |  |
| Service Occupations | 35 | 7.40% |  | 11 | 12.50% |  | 13 | 7.10% |  |  |
| Farming, Forestry, And Fishing Occupations | 11 | 2.30% |  | 0 | 0.00% |  | 2 | 1.10% |  |  |
| Precision Production, Craft, And Repair Occupations | 40 | 8.50% |  | 6 | 6.80% |  | 12 | 6.50% |  |  |
| Operators, Fabricators, And Laborers | 24 | 5.10% |  | 11 | 12.50% |  | 10 | 5.40% |  |  |
| Experienced Unemployed Not Classified By Occupations | 10 | 2.10% |  | 10 | 11.40% |  | 8 | 4.30% |  |  |
| Statistical significance markers: * p<0.1; ** p<0.05; *** p<0.01 | | | | | | |  |  |  |  |

**Table A.3 Distribution of SES indicators stratified by the number of CP locations**

|  | **No pain** | | | **1-2 pain locations** | | | **3+ pain locations** | | |  |
| --- | --- | --- | --- | --- | --- | --- | --- | --- | --- | --- |
|  | **N** | **Mean / %** | **Sd** | **N** | **Mean / %** | **Sd** | **N** | **Mean / %** | **Sd** | **Test** |
| **Childhood SES indicators** |  |  |  |  |  |  |  |  |  |  |
| Fathers education | 454 |  |  | 169 |  |  | 99 |  |  | X2=9.087^*^ |
| less than high school | 153 | 33.70% |  | 54 | 32.00% |  | 44 | 44.40% |  |  |
| high school/GED/some college | 210 | 46.30% |  | 70 | 41.40% |  | 41 | 41.40% |  |  |
| bachelor's degree or more | 91 | 20.00% |  | 45 | 26.60% |  | 14 | 14.10% |  |  |
| Mother education | 477 |  |  | 182 |  |  | 101 |  |  | X2=6.192 |
| less than high school | 123 | 25.80% |  | 48 | 26.40% |  | 34 | 33.70% |  |  |
| high school/GED/some college | 276 | 57.90% |  | 100 | 54.90% |  | 58 | 57.40% |  |  |
| bachelor's degree or more | 78 | 16.40% |  | 34 | 18.70% |  | 9 | 8.90% |  |  |
| Financial level growing up | 482 |  |  | 181 |  |  | 102 |  |  | X2=24.037^**^ |
| A lot better off | 14 | 2.90% |  | 4 | 2.20% |  | 2 | 2.00% |  |  |
| Somewhat better off | 65 | 13.50% |  | 19 | 10.50% |  | 15 | 14.70% |  |  |
| A little better off | 68 | 14.10% |  | 43 | 23.80% |  | 8 | 7.80% |  |  |
| Same as average family | 207 | 42.90% |  | 57 | 31.50% |  | 41 | 40.20% |  |  |
| A little worse off | 85 | 17.60% |  | 39 | 21.50% |  | 20 | 19.60% |  |  |
| Somewhat worse off | 31 | 6.40% |  | 15 | 8.30% |  | 13 | 12.70% |  |  |
| A lot worse off | 12 | 2.50% |  | 4 | 2.20% |  | 3 | 2.90% |  |  |
| Father's occupation | 451 |  |  | 170 |  |  | 98 |  |  | X2=18.274 |
| Managerial And Professional Specialty Occupations | 134 | 29.70% |  | 56 | 32.90% |  | 23 | 23.50% |  |  |
| Technical, Sales, And Administrative Support Occupations | 74 | 16.40% |  | 34 | 20.00% |  | 17 | 17.30% |  |  |
| Service Occupations | 19 | 4.20% |  | 6 | 3.50% |  | 2 | 2.00% |  |  |
| Farming, Forestry, And Fishing Occupations | 74 | 16.40% |  | 21 | 12.40% |  | 10 | 10.20% |  |  |
| Precision Production, Craft, And Repair Occupations | 75 | 16.60% |  | 25 | 14.70% |  | 24 | 24.50% |  |  |
| Operators, Fabricators, And Laborers | 62 | 13.70% |  | 22 | 12.90% |  | 22 | 22.40% |  |  |
| Experienced Unemployed Not Classified By Occupations | 13 | 2.90% |  | 6 | 3.50% |  | 0 | 0.00% |  |  |
| **M1 SES indicators** |  |  |  |  |  |  |  |  |  |  |
| Rate current financial situation | 481 | 6.428 | 2.14 | 178 | 6.36 | 1.924 | 102 | 5.686 | 2.371 | F=5.19^***^ |
| Money to meet needs | 482 |  |  | 181 |  |  | 102 |  |  | X2=6.507 |
| More than enough money | 110 | 22.80% |  | 41 | 22.70% |  | 17 | 16.70% |  |  |
| Just enough money | 274 | 56.80% |  | 99 | 54.70% |  | 53 | 52.00% |  |  |
| Not enough money | 98 | 20.30% |  | 41 | 22.70% |  | 32 | 31.40% |  |  |
| How difficult to pay monthly bills | 483 |  |  | 180 |  |  | 102 |  |  | X2=18.819^***^ |
| Not at all difficult | 173 | 35.80% |  | 57 | 31.70% |  | 15 | 14.70% |  |  |
| Not very difficult | 173 | 35.80% |  | 70 | 38.90% |  | 45 | 44.10% |  |  |
| Somewhat difficult | 115 | 23.80% |  | 43 | 23.90% |  | 33 | 32.40% |  |  |
| Very difficult | 22 | 4.60% |  | 10 | 5.60% |  | 9 | 8.80% |  |  |
| Education | 490 |  |  | 185 |  |  | 105 |  |  | X2=30.578^***^ |
| less than high school | 7 | 1.40% |  | 5 | 2.70% |  | 11 | 10.50% |  |  |
| high school/GED/some college | 251 | 51.20% |  | 90 | 48.60% |  | 61 | 58.10% |  |  |
| bachelor's degree or more | 232 | 47.30% |  | 90 | 48.60% |  | 33 | 31.40% |  |  |
| Income-to-needs ratio | 475 |  |  | 180 |  |  | 101 |  |  | X2=35.269^***^ |
| Affluent | 371 | 78.10% |  | 137 | 76.10% |  | 56 | 55.40% |  |  |
| Adequate-income | 69 | 14.50% |  | 35 | 19.40% |  | 24 | 23.80% |  |  |
| Low-income | 22 | 4.60% |  | 6 | 3.30% |  | 11 | 10.90% |  |  |
| Poor | 4 | 0.80% |  | 1 | 0.60% |  | 3 | 3.00% |  |  |
| Extreme poverty | 9 | 1.90% |  | 1 | 0.60% |  | 7 | 6.90% |  |  |
| Occupation | 480 |  |  | 177 |  |  | 99 |  |  | X2=25.062^**^ |
| Managerial And Professional Specialty Occupations | 220 | 45.80% |  | 89 | 50.30% |  | 40 | 40.40% |  |  |
| Technical, Sales, And Administrative Support Occupations | 133 | 27.70% |  | 47 | 26.60% |  | 21 | 21.20% |  |  |
| Service Occupations | 42 | 8.80% |  | 6 | 3.40% |  | 16 | 16.20% |  |  |
| Farming, Forestry, And Fishing Occupations | 13 | 2.70% |  | 2 | 1.10% |  | 2 | 2.00% |  |  |
| Precision Production, Craft, And Repair Occupations | 33 | 6.90% |  | 14 | 7.90% |  | 9 | 9.10% |  |  |
| Operators, Fabricators, And Laborers | 29 | 6.00% |  | 13 | 7.30% |  | 4 | 4.00% |  |  |
| Experienced Unemployed Not Classified By Occupations | 10 | 2.10% |  | 6 | 3.40% |  | 7 | 7.10% |  |  |
| **M2 SES indicators** |  |  |  |  |  |  |  |  |  |  |
| Rate current financial situation | 485 | 6.713 | 2.028 | 185 | 6.514 | 2.147 | 105 | 5.848 | 2.429 | F=7.266^***^ |
| Money to meet needs | 489 |  |  | 186 |  |  | 105 |  |  | X2=8.6^*^ |
| More than enough money | 170 | 34.80% |  | 55 | 29.60% |  | 31 | 29.50% |  |  |
| Just enough money | 242 | 49.50% |  | 99 | 53.20% |  | 46 | 43.80% |  |  |
| Not enough money | 77 | 15.70% |  | 32 | 17.20% |  | 28 | 26.70% |  |  |
| How difficult to pay monthly bills | 489 |  |  | 186 |  |  | 105 |  |  | X2=23.743^***^ |
| Not at all difficult | 223 | 45.60% |  | 67 | 36.00% |  | 27 | 25.70% |  |  |
| Not very difficult | 161 | 32.90% |  | 68 | 36.60% |  | 36 | 34.30% |  |  |
| Somewhat difficult | 90 | 18.40% |  | 40 | 21.50% |  | 34 | 32.40% |  |  |
| Very difficult | 15 | 3.10% |  | 11 | 5.90% |  | 8 | 7.60% |  |  |
| Education | 489 |  |  | 186 |  |  | 105 |  |  | X2=37.45^***^ |
| less than high school | 4 | 0.80% |  | 6 | 3.20% |  | 11 | 10.50% |  |  |
| high school/GED/some college | 236 | 48.30% |  | 82 | 44.10% |  | 58 | 55.20% |  |  |
| bachelor's degree or more | 249 | 50.90% |  | 98 | 52.70% |  | 36 | 34.30% |  |  |
| Income-to-needs ratio | 483 |  |  | 182 |  |  | 102 |  |  | X2=17.539^**^ |
| Affluent | 292 | 60.50% |  | 101 | 55.50% |  | 44 | 43.10% |  |  |
| Adequate-income | 129 | 26.70% |  | 50 | 27.50% |  | 32 | 31.40% |  |  |
| Low-income | 34 | 7.00% |  | 15 | 8.20% |  | 10 | 9.80% |  |  |
| Poor | 8 | 1.70% |  | 6 | 3.30% |  | 6 | 5.90% |  |  |
| Extreme poverty | 20 | 4.10% |  | 10 | 5.50% |  | 10 | 9.80% |  |  |
| Occupation | 471 |  |  | 175 |  |  | 97 |  |  | X2=33.712^***^ |
| Managerial And Professional Specialty Occupations | 223 | 47.30% |  | 94 | 53.70% |  | 43 | 44.30% |  |  |
| Technical, Sales, And Administrative Support Occupations | 128 | 27.20% |  | 35 | 20.00% |  | 17 | 17.50% |  |  |
| Service Occupations | 35 | 7.40% |  | 10 | 5.70% |  | 14 | 14.40% |  |  |
| Farming, Forestry, And Fishing Occupations | 11 | 2.30% |  | 2 | 1.10% |  | 0 | 0.00% |  |  |
| Precision Production, Craft, And Repair Occupations | 40 | 8.50% |  | 11 | 6.30% |  | 7 | 7.20% |  |  |
| Operators, Fabricators, And Laborers | 24 | 5.10% |  | 15 | 8.60% |  | 6 | 6.20% |  |  |
| Experienced Unemployed Not Classified By Occupations | 10 | 2.10% |  | 8 | 4.60% |  | 10 | 10.30% |  |  |
| Statistical significance markers: * p<0.1; ** p<0.05; *** p<0.01 | | | | | |  |  |  |  |  |

**Table B.1 Baseline Sample Characteristics**

|  | **Attrition (n=6327)** | | | **Participants (n=781)** | | |  |
| --- | --- | --- | --- | --- | --- | --- | --- |
|  | **N** | **Mean / %** | **SD** | **N** | **Mean / %** | **SD** | **Test** |
| **Childhood SES indicators** |  |  |  |  |  |  |  |
| Fathers education | 5379 |  |  | 722 |  |  | X2=9.763^***^ |
| less than high school | 2180 | 41% |  | 251 | 35% |  |  |
| high school/GED/some college | 2248 | 42% |  | 321 | 44% |  |  |
| bachelor's degree or more | 951 | 18% |  | 150 | 21% |  |  |
| Mother education | 5858 |  |  | 760 |  |  | X2=25.718^***^ |
| less than high school | 2035 | 35% |  | 205 | 27% |  |  |
| high school/GED/some college | 3165 | 54% |  | 434 | 57% |  |  |
| bachelor's degree or more | 658 | 11% |  | 121 | 16% |  |  |
| Financial level growing up | 5522 |  |  | 765 |  |  | X2=8.104 |
| A lot better off | 192 | 3% |  | 20 | 3% |  |  |
| Somewhat better off | 652 | 12% |  | 99 | 13% |  |  |
| A little better off | 738 | 13% |  | 119 | 16% |  |  |
| Same as average family | 2344 | 42% |  | 305 | 40% |  |  |
| A little worse off | 973 | 18% |  | 144 | 19% |  |  |
| Somewhat worse off | 432 | 8% |  | 59 | 8% |  |  |
| A lot worse off | 191 | 3% |  | 19 | 2% |  |  |
| Father's occupation | 5538 |  |  | 719 |  |  | X2=31.89^***^ |
| Managerial And Professional Specialty Occupations | 1343 | 24% |  | 213 | 30% |  |  |
| Technical, Sales, And Administrative Support Occupations | 786 | 14% |  | 125 | 17% |  |  |
| Service Occupations | 258 | 5% |  | 27 | 4% |  |  |
| Farming, Forestry, And Fishing Occupations | 699 | 13% |  | 105 | 15% |  |  |
| Precision Production, Craft, And Repair Occupations | 1325 | 24% |  | 124 | 17% |  |  |
| Operators, Fabricators, And Laborers | 1005 | 18% |  | 106 | 15% |  |  |
| Experienced Unemployed Not Classified By Occupations | 122 | 2% |  | 19 | 3% |  |  |
| **M1 SES indicators** |  |  |  |  |  |  |  |
| Rate current financial situation | 5470 | 6.1 | 2.2 | 761 | 6.3 | 2.1 | F=5.453^**^ |
| Money to meet needs | 5487 |  |  | 765 |  |  | X2=24.868^***^ |
| More than enough money | 849 | 15% |  | 168 | 22% |  |  |
| Just enough money | 3112 | 57% |  | 426 | 56% |  |  |
| Not enough money | 1526 | 28% |  | 171 | 22% |  |  |
| How difficult to pay monthly bills | 5484 |  |  | 765 |  |  | X2=9.254^**^ |
| Not at all difficult | 1498 | 27% |  | 245 | 32% |  |  |
| Not very difficult | 2079 | 38% |  | 288 | 38% |  |  |
| Somewhat difficult | 1578 | 29% |  | 191 | 25% |  |  |
| Very difficult | 329 | 6% |  | 41 | 5% |  |  |
| Education | 6315 |  |  | 780 |  |  | X2=112.279^***^ |
| less than high school | 658 | 10% |  | 23 | 3% |  |  |
| high school/GED/some college | 3831 | 61% |  | 402 | 52% |  |  |
| bachelor's degree or more | 1826 | 29% |  | 355 | 46% |  |  |
| Income-to-needs ratio | 5354 |  |  | 756 |  |  | X2=57.804^***^ |
| Affluent | 3254 | 61% |  | 564 | 75% |  |  |
| Adequate-income | 1243 | 23% |  | 128 | 17% |  |  |
| Low-income | 455 | 8% |  | 39 | 5% |  |  |
| Poor | 132 | 2% |  | 8 | 1% |  |  |
| Extreme poverty | 270 | 5% |  | 17 | 2% |  |  |
| Occupation | 6065 |  |  | 756 |  |  | X2=49.74^***^ |
| Managerial And Professional Specialty Occupations | 2130 | 35% |  | 349 | 46% |  |  |
| Technical, Sales, And Administrative Support Occupations | 1667 | 27% |  | 201 | 27% |  |  |
| Service Occupations | 541 | 9% |  | 64 | 8% |  |  |
| Farming, Forestry, And Fishing Occupations | 120 | 2% |  | 17 | 2% |  |  |
| Precision Production, Craft, And Repair Occupations | 678 | 11% |  | 56 | 7% |  |  |
| Operators, Fabricators, And Laborers | 599 | 10% |  | 46 | 6% |  |  |
| Experienced Unemployed Not Classified By Occupations | 330 | 5% |  | 23 | 3% |  |  |
| **Demographic characteristics** |  |  |  |  |  |  |  |
| Sex | 4182 |  |  | 781 |  |  | X2=1.025 |
| Male | 1965 | 47% |  | 351 | 45% |  |  |
| Female | 2217 | 53% |  | 430 | 55% |  |  |
| Ethnicity | 5488 |  |  | 766 |  |  | X2=10.392^***^ |
| White | 4931 | 90% |  | 717 | 94% |  |  |
| non-White | 557 | 10% |  | 49 | 6% |  |  |
| Age | 6268 | 47 | 13 | 781 | 45 | 11 | F=10.492^***^ |
| Marital status | 6322 |  |  | 781 |  |  | X2=25.876^***^ |
| Married | 4108 | 65% |  | 558 | 71% |  |  |
| Divorced | 868 | 14% |  | 94 | 12% |  |  |
| Never married | 820 | 13% |  | 99 | 13% |  |  |
| Separated | 185 | 3% |  | 16 | 2% |  |  |
| Widowed | 341 | 5% |  | 14 | 2% |  |  |
| **Health characteristics** |  |  |  |  |  |  |  |
| Physical activity | 5501 | 4.6 | 1.3 | 761 | 4.9 | 1.0 | F=33.123^***^ |
| Smoking status | 6322 |  |  | 781 |  |  | X2=50.869^***^ |
| Current smoker | 1520 | 24% |  | 101 | 13% |  |  |
| Ex-smoker | 3298 | 52% |  | 449 | 57% |  |  |
| non-Smoker | 1504 | 24% |  | 231 | 30% |  |  |
| Drinking status | 6327 |  |  | 781 |  |  | X2=7.273^**^ |
| Moderate + Drinker | 3886 | 61% |  | 469 | 60% |  |  |
| Light Drinker | 1766 | 28% |  | 247 | 32% |  |  |
| non-Drinker or Rarley Drink | 675 | 11% |  | 65 | 8% |  |  |
| Multimorbidity | 5542 |  |  | 766 |  |  | X2=5.768^**^ |
| <2 | 2461 | 44% |  | 376 | 49% |  |  |
| 2+ | 3081 | 56% |  | 390 | 51% |  |  |
| Statistical significance markers: * p<0.1; ** p<0.05; *** p<0.01 | | |  |  |  |  |  |
